# Charting a “Green Path” for Recovery from COVID-19

**DOI:** 10.1007/s10640-020-00479-0

**Published:** 2020-08-04

**Authors:** Samson Mukanjari, Thomas Sterner

**Affiliations:** 1 Environment for Development and Department of Economics, University of Gothenburg, Box 640, 405 30 Gothenburg, Sweden; 2 Public and Environmental Economics Research Centre, University of Johannesburg, Johannesburg, South Africa; 3 Department of Economics, University of Gothenburg, Box 640, 405 30 Gothenburg, Sweden

**Keywords:** Carbon emissions, Climate change, Climate policy, COVID-19, Green recovery, Stock returns, G14, Q40, Q5

## Abstract

Should the economic recovery from the 2019 novel coronavirus disease (COVID-19) be green? The current crisis is so severe that we should not take the answer for granted. It requires serious thought and we start by reviewing some arguments for and against a green approach. A crucial element is of course to see how different industries fare in the current crisis. Our empirical contribution is to examine daily stock returns for firms from the STOXX Europe 600 index. We find that firms with higher carbon intensities experienced significantly large decreases in stock values particularly those within the crude petroleum extraction, air transport and coke and refined petroleum industries. Our tentative conclusion is that efforts to revitalize the economy should avoid subsidizing stranded assets and instead target the industries of the future. However, identifying these will not necessarily be easy. We find, for example, that having an official ESG “climate change policy” has no effect on firm performance during the pandemic. We suggest possible ways of designing a new form of more informative index.

## Introduction

The 2019 novel coronavirus disease (COVID-19) crisis is such a major event that in many countries debate and criticism of authorities has been muted. People stand behind their political leaders irrespective of their party affiliations and even independently it seems of how sensible the policies are. Quite a few leaders see an opportunity here and are consolidating their power and weakening the opposition. In some cases, there are clear attacks on democracy. There has also been quite a backlash against environmentalism. In many countries, fuel or carbon taxes have been (temporarily?) reduced and environmental regulations rolled back (Abnett 2020; Carrington 2020; Helm 2020; McVeigh 2020; Milman and Holden 2020; Spring 2020). There are many who think we should focus on saving the big automobile companies, oil companies and airlines and that the size of this emergency means we cannot afford the luxury of thinking of environmental issues.

There has been intense debate in the media about the impact of the pandemic on ongoing climate initiatives across the world. There is a real risk that climate change may be relegated to the periphery as countries focus on economic revival necessary to create employment and reduce poverty.^1^ Prior to the outbreak of the pandemic, environmental issues made up the top five business risks for 2020 (World Economic Forum 2020). The year 2020 promised to be a turning point for climate action. Countries were expected to announce new pledges to reduce greenhouse gas (GHG) emissions ahead of the Glasgow summit in November 2020. However, the most important UN Framework Convention on Climate Change (UNFCCC) Conference of the Parties (COP) to take place since the 2015 Paris Agreement has been postponed due to COVID-19, which is now declared a pandemic. The pandemic has had devastating effects across the world and has dramatically altered the way in which societies function. Some of the environmental effects of the pandemic have been a reduction in CO_2_ emissions as economic activity came to a sudden halt (Carbon Brief 2020; IEA 2020; Le Quéré et al. 2020).

Pollution levels have fallen drastically for conventional pollutants like soot and NOx and the pandemic is on track to trigger the largest ever annual drop in global CO_2_ emissions. This drop for carbon is however nowhere near the declines for local pollutants, but just in line with what would be required every year to reach the goals in the Paris Agreement. Furthermore, carbon is a stock pollutant and we are discussing emissions levels—the carbon content of the atmosphere is still rising fast. Atmospheric carbon dioxide measured at the Mauna Loa Observatory just recorded 417.1 parts per million for May 2020, the highest monthly reading ever recorded (NOAA and Scripps 2020). This suggests quite strongly that we do not want to address climate change with reductions in economic output as our main instrument. It has to be done through changing technology in energy and other sectors—worldwide—and for this to happen strong policies are needed across the whole world.

There has been much debate on the existence of tipping points in the physical climate system. Suddenly, it is dawning on us that we may already be at a socio-economic tipping point. The combination of a pandemic-induced public health and economic crisis has created a situation where the customary forecasting of GDP and other vital signs of the economy is clearly unable to come up with any reliable forecasts (Baker et al. 2020). The future is unknown and it is up to us to shape. Compounding the health and economic uncertainty is the political response uncertainty which is arguably the most volatile. Will the world come together to solve joint challenges or will the world economic order, free trade, peace and democracy be severely damaged and thus reinforcing the precarious downward spiral for the economy?

In Sect. 2, we provide a number of arguments from the literature for and against making the recovery out of the current crisis “green”—i.e. tying together our response to the COVID-19, economic and climate crises. Section 3 provides some background thoughts on studying the current crisis and seeing it through the lens of financial markets. Section 4 describes our methodology and data—how we use financial market data (daily stock returns for firms from the STOXX Europe 600 index) in an event study analysis of the effects of the current crisis between- and within-industries. Section 5 presents the results and Sect. 6 concludes.

## Should the Recovery be Green?

It comes naturally for many environmentalists to argue for a green recovery (Barbier 2020; Bozuwa et al. 2020; Kåberger and Sterner 2020; Schumacher et al. 2020). A significant movement within business has also expressed demands for a green transition (see for example UK Stakeholders for Sustainable Development and Global Compact Network UK 2020). With hundreds of thousands of casualties from COVID-19 and a severe economic crisis, many politicians are literally overwhelmed. Yet the journal of Environmental and Resource Economics—and many others choose to write about a third crisis: the climate. Is it fundamentally a good idea to combine and discuss these issues and to analyze them together? Let us pause to think whether the economic recovery should be climate friendly. There are a number of respectable arguments against a strong green focus:

- The first and strongest is that this is another kind of crisis and what is needed is conventional stimulus to save restaurants, consultancies, hairdressers, musical theatres—and yes even transport companies. Otherwise, this health and economic crisis will tear apart the social and economic fabric of society. In fact, the magnitude of this crisis is such that it will require policies out of the ordinary when it comes to fiscal stimulus and monetary policy. This is too big to combine with other policies. We can take one step further and particularly focus on developing countries where lock-down is synonymous with starvation and where the state has very limited resources. In the tracks of the pandemic we risk mass starvation and on top of that a chaos that can topple the current order, maybe worsen the epidemic, maybe destroy democratic and economic institutions. In that perspective, the first and maybe only order for the day should be damage control and saving employment as well as avoiding corruption and trying, as much as possible to spread the benefits of assistance fairly.
- A second argument is that climate policy needs general, long run policy and is not helped by rapid improvisations under the pressure from a disaster. Of course, if we save companies we can (perhaps) impose conditions and require auto and airline manufacturers to promise to be greener.^2^ But why not general green policies instead?
- We risk sounding like the general who always says we need more money for the army or the priest who always says this is a good time to reflect on our inevitable death and the importance of the afterlife. The general lesson is that we lose credibility if we always harp on the same tune.

There are however also very strong arguments why the crisis response should be “green”:

- Some argue that we should not be in such a hurry to get “back to normal”. It is this “normal” they see as the root cause of the climate crisis. Certainly, we know how difficult it is to get any meaningful climate policy enacted during “normal” times. The now cancelled, or postponed, Conference of the Parties in Glasgow would have been COP ***26*** and many argue that this essentially means 25 years of negotiation severely deficient in actual results as far as global policies are concerned. More and more articles are written. More and more negotiators travel and spend days and nights negotiating—but the stock of carbon keeps growing, in many cases even the emissions continue to grow which means that the stock is growing faster and faster.
- People also point to the gross “local” pollution levels in cities and even giant areas as witnessed by the Asian Brown Cloud (UNEP and C^4^ 2002). Finally, the world order with fragmented supply chains has implied a level of transportation that is environmentally problematic and leaves us vulnerable to disturbances. This was illustrated early in this pandemic when the shutdown in Wuhan caused industries all over the world to close for lack of parts.
- Another argument for specifically green policies is that we do face several serious crises. The political system is only capable of handling a certain number of policy items and thus the crises must be dealt with simultaneously if this is possible. At least, one should prioritize those measures that do happen to address both problems!
- A related argument focuses on the size of the spending necessary to solve the short-run economic crisis. The economic response by the rich countries has already surpassed the response to the 2007–2009 financial crisis with the fiscal support by the G20 countries estimated to be $7 trillion as of May 29, 2020 (Segal and Gerstel 2020). As the scale of the pandemic unfolds, it is clear that the current spending will amount to many years of normal discretionary spending or ordinary budgetary reforms. Even countries in southern Europe are already struggling with limited fiscal space and the solidarity and cohesion of the European Union is put to the test as its less wealthy members press for joint funding of recovery measures.
- In the developing world, the ability to effectively deal with the current crisis is already severely hampered by a paralyzing lack of funds. It is vital for democracy and for long run recovery that this money is spent in a way that is efficient, fair, non-corrupt and of lasting value. If it is not spent fairly we will have severe social unrest. If all is spent saving airlines, oil- and car-companies, some of which will anyway be tomorrow’s stranded assets, we will have wasted an opportunity to transition to an environmentally sustainable future. At the same time, we must realize that there is a very real risk that exactly this will happen. Real economic policy is, in many countries, heavily influenced by lobbyists and this will tend to be reinforced in the acute struggle during a pandemic crisis. When very large economic transactions are to be decided outside of the regular budgetary and government procedures in a very limited period of time, the advantages of incumbency will be strongly amplified. The biggest of companies already have material prepared to press their cases to government ministers. In the energy sector, the fossil interests are very concentrated to large companies while companies with new technology, startups, renewables energy and efficiency investments are much more dispersed and have less coordinated lobbying power.

The greenness of the economic recovery packages is not a black or white issue—it is partly a matter of timing. The immediate response is likely be more about stabilizing the economy but gradually the focus should move over to green. Thinking systematically about the appropriate policy packages, a number of different arguments have been identified. A policy should ideally have a number of characteristics and there may well be some trade-off between them. Desirable are speed of implementation, fairness in distributional consequences, size of multiplier effect and potential to contribute to the creation of long-term sustainable value.

The so called Stern–Stiglitz report focuses in particular on the tradeoff between economic multiplier effects, speed of implementation and climate impact potential (Hepburn et al. 2020). They point out that the Global Financial Crisis showed that green stimulus policies often have advantages over traditional fiscal stimulus. This applies to renewable energy investments, which are attractive both in the short and in the long run. They have a high multiplier effect. This is a short run concern and renewables typically generate many jobs in the short run during their construction phase just when employment is needed. In the long run, such energy investments actually use less labour and typically no raw materials or fuel for operation and maintenance. This is another way of saying that they are cheaper and economically more efficient which is good for long run growth. The same applies to many projects related to increased energy efficiency for instance in residential or commercial buildings, such as insulation retrofits or clean energy infrastructure. This type of green investments might have not only positive multiplier effects but also technological externalities if learning curves are steep and there are increasing returns to scale as it seems in for instance photovoltaic technologies (Acemoglu et al. 2012).

Hepburn et al. (2020) survey a number of experts in central banks and other institutions to identify a number of policies that combine a large long-run multiplier and strongly positive impact on climate. These include connectivity infrastructure, general R&D spending, education investment, clean energy infrastructure and clean energy R&D spending. Further positive policy options included healthcare investment and worker retraining. Other policies were characterized by a tradeoff. They were either high on climate impact (green spaces and natural infrastructure) or high multiplier and other positive characteristics such as speed of implementation—for instance liquidity support for households, start-ups, and SMEs, targeted or direct cash transfers. On the other hand, airline bailouts did poorly by all metrics.

One factor of particular importance for the selection and design of policy instruments is the economic context. Since the pandemic, we have witnessed a dramatic fall in GDP. In many countries this translates into increased unemployment, although this factor is somewhat mediated in for example European welfare states where insurance schemes, furloughs and public measures to avoid open unemployment are undertaken. There is also a rapid reduction in use of fossil fuels, which in turn leads to big reductions in local pollution and some reduction in carbon emissions (although, as mentioned, for a stock pollutant like atmospheric carbon the effect is minute). Finally, it also leads to rapid fall in fossil prices. This last factor deserves some particular attention.

The price of oil in particular fell by more than two-thirds since January. At the same time, the US is becoming a more important fossil fuel producer and under the current President, the United States has taken unprecedented steps to collaborate with OPEC instead of criticizing its cartel behavior. Even the *Financial Times* speaks incredulously of the US policies and describes Trump as “essentially becoming the de facto OPEC president” (Brower et al. 2020). Not only has Trump tried to mend the fences between Saudi Arabia and Russia. He has also taken steps to reduce US oil extraction and support oil prices as part of an enlarged OPEC of all oil asset owners. In spite of these efforts, US crude oil futures prices went *negative* for the first time ever.^3^ There is a clear risk, that economies seeking to kick-start and save employment in a period of very cheap oil may be tempted to choose to revert from the green transition and rely on old-school technologies—thereby risking of course ending up with stranded assets in a few years.

Carbon prices are key drivers and have been responsible for pushing coal out of the European electricity mix (− 12% of emissions in 2019 alone). Renewables are the fastest growing source of energy currently (in fact maybe the only source that is not shrinking currently) (IEA 2020; Kåberger and Zissler 2020).

Today’s low oil prices provide governments with an opportunity for a policy that is good for the economic recovery and for the climate. It is the opposite of OPEC’s strategy. Instead of helping oil owners secure their rents, most countries should raise taxes on fossil fuels. This applies of course in particular to oil (or fossil) importing countries. One possibility is to create a new additional floating tax that compensates for movements in oil prices. The target might for instance be to maintain the consumer prices at the levels seen in the beginning of the year 2020. With current prices, this could generate revenues of 30 cents per liter, or the equivalent of around 100 billion Euros a year (Sterner et al. 2020). Considering the struggle to raise EU funding for the European Green Deal, such a sum would not be insignificant.

The increase in excise duties will maintain incentives for people to limit their fossil fuel consumption and continue to invest in low carbon alternatives. Without such correction, a post-crisis rebound effect—an increase in emissions—is likely. Such a rebound would be totally counter to the efforts to deal with climate change and moreover imply a clear risk that new investments in both energy production and other energy consuming sectors turn out to be stranded assets lowering future growth. Keeping the price of fossil products to their pre-crisis levels is the best way to inform the consumers, investors and other agents of the economy and to support current emission reduction efforts and transition efforts.

In developing countries, the dire need for funds may push policy makers in this direction. India has for instance already raised the excise taxes on transport fuels like gasoline. The excise duties on these fuels have been increased by 3 rupees per liter, which is estimated to give an additional revenue to the Indian state of 400 billion rupees (over US$5 billion). The main purpose is likely more budgetary than climate related but the effect will of course be a decrease in emissions. If enough importing countries follow the same policy it may contribute to reducing demand, keeping international (pre-tax) oil prices low. This can disincentivize new fossil investments and provide positive incentives for renewables thus enabling a positive spiral with more consumer taxes, continued low producer prices of fossil fuel and incentives for renewables.

As a response to the climate crisis and the risk of stranded assets, there is a good deal of demand for sustainable investment opportunities. Individuals, banks and pension funds seek to ensure that their portfolios are “green” or at least to avoid the risks of stranded assets. This is however more difficult than it might first appear. Giant fossil companies may be relatively easy to identify but there are numerous large companies that have a plethora of activities that may be more or less related to the energy sector and more or less carbon intense. How is the investor supposed to identify these?

## COVID-19 and the Stock Market

The COVID-19 outbreak has affected every economic sector as the health crisis quickly evolved into an economic crisis. Some of its impacts have been witnessed on stock markets across the world. The effects of COVID-19 on the broader stock market and growth expectations have been studied by Baker et al. (2020a, b), Gormsen and Koijen (2020), and Ramelli and Wagner (2020). Despite the clear environmental effects of the pandemic, little attention has been devoted to understanding how the outbreak affected the stock market valuation of publicly traded firms depending on their level of carbon emissions or carbon-specific commitments. While the demand for energy itself has largely fallen in response to the outbreak, preliminary evidence suggests the effect has been heterogeneous across the different energy sources. Whereas fossil fuel energy demand has fallen during the course of the pandemic, the demand for renewable energy has increased in many countries (IEA 2020; Kåberger and Zissler 2020).

The demand response for the different energy sources during the pandemic provides important insights for policy makers looking for early signs of the likely effects of COVID-19 on the climate momentum. There have been significant concern that the COVID-19 crisis may present a setback to the recent progress made by renewable energy. In this paper, we use stock price reactions to gain valuable insights on the performance differences between- and within-industries that comprise of firms possessing different levels of carbon intensity and climate responsibility.^4^ There is an emerging and growing literature suggesting that carbon emissions significantly affect stock returns (Bolton and Kacperczyk 2020; Garvey et al. 2018). In the present study, we focus on European firms and use the COVID-19 outbreak to understand whether past climate responsibility (defined as having lower emissions or explicit climate-specific commitments) is rewarded and previous dirtier deeds punished during crisis periods. Using stock prices has an advantage in that asset prices capture current expectations. The stock market thus gives us a continuously updated summary of investor beliefs regarding the economic impacts of the pandemic. However, it is important to note that as far as energy demand and stock price reaction during the pandemic period are concerned, there are several confounding factors, which make it difficult to attribute any changes solely to the pandemic. Examples include the oil price war between OPEC and Russia followed by the agreement within the framework of the enlarged OPEC + coalition, mild winter in Europe and North America and the increasing renewable energy generating capacity.^5^ Nevertheless, the COVID-19 pandemic remains the most notable event during this period in terms of the magnitude of its impact on every economic sector.

The coronavirus disease emerged from Wuhan, China at the beginning of January 2020 before spreading to the rest of the world. Within Europe, Italy became the first country to be significantly affected before the disease gradually made its way into the rest of Europe and North America. The pandemic then spread to Asia, Latin America and finally Africa. With the partial exception of Sweden, most European countries reacted by closing their borders while simultaneously introducing national lockdowns characterized by severe restrictions on human movement. The pandemic has thus affected the economy through several different paths, the importance of which may vary between countries and industries. One of the paths is direct as when workplaces close due to infections or risk of infections. Another series of paths are the indirect ones that relate to reduced purchasing power and or increased caution on the part of consumers and various other macro-economic feedbacks. Finally, one path is the Chinese lockdown. There has been plenty of discussion concerning the sensitivity of the global supply chains that were disrupted when China locked down.^6^

In this paper, we analyze the relative performance of different industries and firms within different industries across 18 European countries. We focus on a sample of European firms since the EU has made significant progress on policies aimed at reducing greenhouse gas (GHG) emissions. On the contrary, the US has seen most of the climate initiatives that existed before the Trump Administration rolled back.^7^ We first provide a snapshot of the reaction of all industries over the period January to March 2020 before focusing our analysis on within-industry differences in performance. Our interest is on within-industry differences in terms of carbon exposure and climate responsibility. In the next sections, we present the estimation strategy and data followed by the main empirical results and a discussion of the results.

## Empirical Approach and Data

Our empirical approach compares average stock performance of portfolios comprising firms that differ with respect to their industry and carbon intensity. The approach has two stages, the first of which uses the event study approach to estimate abnormal returns during the pandemic period while the second stage investigates the cross-sectional determinants of the abnormal returns. The key assumptions for event studies are that markets are efficient, the event’s timing is exogenous, and the event is unanticipated. The COVID-19 outbreak provides an unwelcome natural experiment to assess how the stock market values firms with different carbon intensities and climate-specific commitments. In order to examine the stock’s abnormal price behavior, a normal return for the stock during the period preceding the event period must be established. The abnormal return is defined as the difference between the normal return for the firm and the firm’s actual return during the event period. The normal return within short-term event studies is estimated using asset pricing models. The main approaches are the market model (Sharpe 1963) and the single-factor model based on the Capital Asset Pricing Model (CAPM) (Sharpe 1964). The unconditional CAPM is given as^8^

1 $$R_{ijt} - \, R_{ft} = \, a_{i} + \, b_{i} \left[ {R_{mt} - \, R_{ft} } \right] + \epsilon_{ijt}$$

where *R*_*ijt*_ is the daily stock return for firm *i* in industry *j*, *R*_*mt*_ is the return in the overall market, *R*_*ft*_ is the risk-free interest rate and *ϵ*_*ijt*_ is the error term with E[*ϵ*_*ijt*_] = 0 and Var[*ϵ*_*ijt*_] = $\upsigma_{ij}^{2}$. The unknown firm-specific parameters of the model to be estimated using ordinary least squares over the estimation period are denoted by *a*_*i*_, *b*_*i*_ and $\upsigma_{ij}^{2}$. The estimation period for the normal return is from January to December 2019 while the event period consists of the period January to March 2020.

Several studies (Fama and French 1992, 1993, 1996) show that the three-factor model by Fama and French (1993) possess more explanatory power compared to the single-factor model. The three-factor model includes two additional factors to explain the excess returns (*R*_*ijt*_ *−* *R*_*ft*_) and is given as

2 $$R_{ijt} - \, R_{ft} = \, a_{i} + \, b_{i} \left[ {R_{mt} - \, R_{ft} } \right] + \, s_{i} SMB_{t} + \, h_{i} HML_{t} + \epsilon_{ijt}$$

where *SMB*_*t*_ is the size factor measured as the difference between the returns of diversified portfolios comprising stocks of small firms and big firms at the end of day *t*. *HML*_*t*_ is the value factor measured as the difference between the returns of diversified portfolios comprising stocks of firms with a high book-to-market equity ratio and firms with a low book-to-market equity ratio at the end of day *t*. *ϵ*_*ijt*_ is the error term with E[*ϵ*_*ijt*_] = 0 and Var[*ϵ*_*ijt*_] = $\upsigma_{ij}^{2}$. The unknown firm-specific parameters of the model to be estimated using ordinary least squares over the estimation period are denoted by *a*_*i*_, *b*_*i*_, *s*_*i*_, *h*_*i*_ and $\upsigma_{ij}^{2}$.

**Fig. 1:**
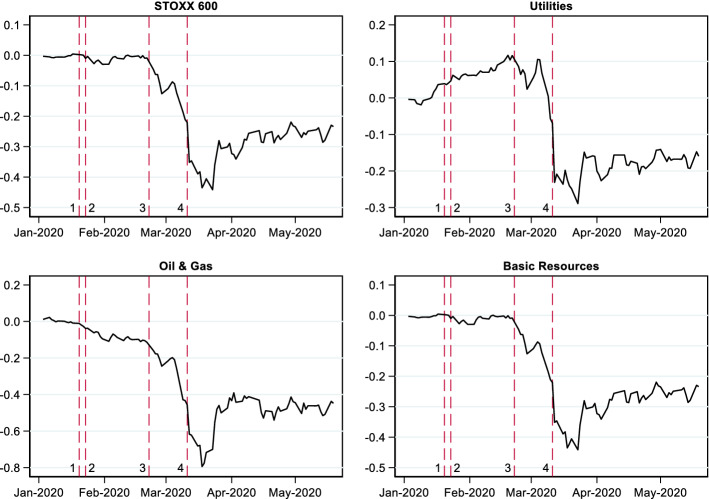
Stock market response. *Note*: This figure plots the cumulative log returns to the STOXX 600 index and its constituent indices from January until the end of May 2020. The dotted lines marks days with significant events. (1) 17/01/2020: Wuhan lockdown, (2) 20/01/2020: China confirms human-to-human transmission of coronavirus, (3) 22/02/2020: Italy quarantine and (4) 11/03/2020: US imposes a travel ban on the EU and the World Health Organization (WHO) declares COVID-19 a pandemic

The inclusion of additional factors is important when one wants to eliminate the impact of factors unrelated to the effects under investigation. In our case, raw returns are preferable given that controlling for the market index may unintentionally absorb the desired event effect. Very large system-changing events such as the one under consideration may also directly affect the market index as shown in Fig. 1 (see also Langer and Lemoine 2019). Doing event studies for such dramatic all-encompassing changes as this pandemic raises some issues that are different from traditional event studies, which mostly focus on firm- or industry-specific events such as earnings announcements or regulatory events (see Kothari and Warner 2007; MacKinlay 1997).

From Eqs. (1) and (2), the abnormal return for firm *i* in industry *j* on day *t* is given as $\widehat{AR}_{ijt} = \left( {R_{ijt} - R_{ft} } \right) - \hat{a}_{i} - \hat{b}_{i} \left[ {R_{mt} - R_{ft} } \right]$ and $\widehat{AR}_{ijt} = \left( {R_{ijt} - R_{ft} } \right) - \hat{a}_{i} - \hat{b}_{i} \left[ {R_{mt} - R_{ft} } \right] - \hat{s}_{i} SMB_{t} - \hat{h}_{i} HML_{t}$ where the coefficients $\hat{a}_{i}$, $\hat{b}_{i}$, $\hat{s}_{i}$ and $\hat{h}_{i}$ are the OLS estimates of *a*_*i*_, *b*_*i*_, *s*_*i*_ and *h*_*i*_. For a sufficiently long estimation period, the estimated abnormal returns will be approximately normally distributed with a zero mean and variance $\upsigma_{ij}^{2}$ under the null hypothesis that the abnormal returns are zero.

From the estimated abnormal returns, the cumulative abnormal return $\left( {CAR_{ijt} } \right)$ is generated as $CAR_{{ij,\left( {t_{0} ,t_{1} } \right)}} = \mathop \sum \nolimits_{{t = t_{0} }}^{{t_{1} }} \widehat{AR}_{ijt}$, where $t_{0}$ is the first day of the event window and $t_{1}$ the last day. The cumulative average abnormal return $\left( {CAAR_{{j,\left( {t_{0} ,t_{1} } \right)}} } \right)$ for a sample of *N* firms in industry *j* over the event window is given as $CAAR_{{j,\left( {t_{0} ,t_{1} } \right)}} = \frac{1}{N}\mathop \sum \nolimits_{i = 1}^{N} CAR_{{ij,\left( {t_{0} ,t_{1} } \right)}}$.

To investigate the cross-sectional determinants of the abnormal returns, we regress the *CARs* on firm characteristics and measures of firm-level carbon intensity and climate responsibility. Specifically, we estimate variants of the following cross-sectional regression:

3 $$CAR_{ijk} = \alpha_{0} + \alpha_{1} Carbon\,Intensity_{ijk} + \alpha_{2} X_{ijk} + \gamma_{j} + \, \zeta_{k} + v_{ijk}$$

where *CAR*_*ijk*_ is the cumulative abnormal return of firm *i* in industry *j* and country *k*. *Carbon Intensity*_*ijk*_ is carbon intensity for firm *i* in industry *j* and country *k*. *X*_*ijk*_ includes a set of firm level characteristics (firm size, profitability and leverage). γ_*j*_ and ζ_*k*_ are industry and country fixed effects and *v*_*ijk*_ is an error term.^9^ The parameter α_1_ is estimated from changes in carbon intensity within the same industry across countries, compared to other industries in a given country. Any confounding variable that has a common effect on *CARs* across all industries in the same country are therefore controlled for. The standard errors are clustered at the country level as well as both the industry and country level.

Our main variable of interest is *Carbon Intensity*_*ijk*_ which represents GHG emissions normalized by the firm’s market capitalization on the day before the event window. GHG emissions are measured as the sum of Scope 1 and 2 emissions. Scope 1 captures direct emissions from production while Scope 2 includes indirect emissions from consumption of purchased electricity, heat, or steam. Scope 1 and 2 emissions have been more widely reported while Scope 3 emissions are less disclosed for many companies and of course harder to measure. Scope 3 emissions includes other indirect emissions that arise from sources not owned or controlled by the company.

In addition to carbon intensity, we also make use of two alternative measures of climate responsibility, namely *Climate Change Policy* and *Environmental Performance*. *Climate Change Policy* is a dummy variable equal to 1 for firms that have outlined their intention to help reduce global emissions of the GHGs through their ongoing operations or the use of their products and services, and zero otherwise. Examples might include efforts to reduce GHG emissions, improve energy efficiency, derive energy from cleaner fuel sources as well as investment in product development to reduce emissions generated or energy consumed in the use of the firm’s products etc. The second climate responsibility measure, *Environmental Performance*, is a dummy variable equal to 1 for firms in the top quartile of environmental performance, and zero otherwise. This variable is derived from Sustainalytics Environment Percentile which gives the industry percentile rank for the firm’s management of its environmental record. For the top 1% the percentile is 99%; for the bottom 1% the percentile is 1%. Environmental performance is determined by the level of environmental preparedness and disclosure in addition to environmental controversies.

The vector *X*_*ijk*_ includes accounting variables such as *Firm Size*_*ijk*_ (natural log of market capitalization for firm *i* in industry *j* and country *k*), *Leverage*_*ijk*_ (financial leverage calculated as average total assets divided by average total common equity and a measure of a firm’s debt level) and return on assets (*Profitability*_*ijk*_)—a measure of a firm’s profitability.

We also control for the stringency of containment policies. For this, we use the Stringency Index developed by Hale et al. (2020). The index records the strictness of ‘lockdown style’ policies that primarily restrict people’s behaviour. This data is obtained from Oxford COVID-19 Government Response Tracker (OxCGRT), which collects publicly available information on 17 indicators of government responses. The Stringency Index is available at the daily frequency for the period starting 1 January 2020 and is constructed from eight of the policy indicators, which record information on containment and closure policies, such as school closures and restrictions in movement. For our purposes, we average the index for each country over the period January to March which is our event period.

Daily stock prices and firm characteristics of all the STOXX 600 index constituents as of December 31, 2019, is obtained from Bloomberg and Thomson Reuters. The security-level CO_2_ emission data is obtained from Thomson Reuters ASSET4. We calculate stock returns using Bloomberg stock prices adjusted for dividends, stock splits, spin-offs and other capital events. The European market factor and the risk free interest rate are obtained from the website of Kenneth French.^10^ The risk free rate is the US 1 month T-bill rate. All the returns are in US dollars. The analysis is limited to stocks that trade continuously during the sample period and have non-missing estimation period returns data for at least 100 trading days. In order to handle non-trading stocks, we calculate trade-to-trade returns from the non-missing price days. If a stock has a missing price, the corresponding market-index return is added to the next non-missing price day’s index return in order to calculate the trade-to-trade abnormal returns. Table 1 shows the summary statistics for all the firms in our sample. The average firm realized raw returns of − 32% during the event period. From Table 1, the average carbon intensity for the firms is 0.19 with a standard deviation of 0.85 showing a large variability. The number of stocks and Stringency Index for each country are given in Table 6 in the “Appendix”. UK firms constitute 25% of the sample. The Stringency Index value over the event period is lowest for Sweden (6.17) and highest for Italy (39.52). This corresponds quite well to a popular perception of confinement stringency in these two countries. We note however, that this variable is also correlated with how early, and how hard the two countries were hit by the initial onslaught of the pandemic.

**Table 1:** Summary statistics

	N	min	p25	Mean	p50	p75	max	SD
Raw returns (RAW)	595	− 91.17	− 44.53	− 31.64	− 31.46	− 19.01	46.99	19.06
CAPM-adjusted returns (CAPM-adj)	595	− 130.46	− 17.88	− 5.04	− 3.33	9.70	57.38	21.99
Fama–French–adjusted returns	595	− 120.58	− 14.30	− 3.74	− 3.17	9.59	53.98	19.13
*Firm characteristics*								
Firm size	595	20.90	22.50	23.23	23.06	23.84	26.50	0.99
Profitability	594	− 19.43	1.50	6.34	4.27	8.29	236.78	12.35
Leverage	585	1.01	1.97	5.51	2.64	4.42	241.22	12.14
Carbon intensity	592	0.00	0.00	0.19	0.01	0.05	10.16	0.77
Climate change policy	589	0.00	1.00	0.75	1.00	1.00	1.00	0.43
Environmental performance	464	0.00	0.00	0.25	0.00	0.50	1.00	0.43
*National lockdown stringency*								
Stringency index	18	6.17	15.51	19.07	18.50	20.43	39.52	6.54

This table shows summary statistics of the variables used in the analysis. The sample consists of STOXX 600 index constituents. Stock prices and firm characteristics data is obtained from Bloomberg and Thomson Reuters. The cumulative raw returns are compounded returns while the CAPM-adjusted returns and Fama–French-adjusted returns are computed using Eqs. (1) and (2) respectively. The variable *Firm Size* is the natural log of market capitalization, *Leverage* is financial leverage calculated as average total assets divided by average total common equity and *Profitability* is the return on assets—a measure of a firm’s profitability. The variable *Carbon Intensity* is represented by GHG emissions normalized by the firm’s market capitalization on the day before the event window. GHG emissions are measured as the sum of Scope 1 and 2 emissions. *Climate Change Policy* is a dummy variable equal to 1 for firms that have outlined their intention to help reduce global emissions of the GHGs through their ongoing operations and or the use of their products and services, and zero otherwise. *Environmental Performance* is a dummy variable equal to 1 for firms in the top quartile of environmental performance, and zero otherwise. The *Stringency Index* captures the strictness of country-level ‘lockdown style’ policies that primarily restrict people’s behavior

Figure 1 shows the cumulative return on the STOXX 600 index and some of its constituent indices. There does not seem to be any strong reaction to the imposition of the lockdown in Wuhan on January 17 and China’s subsequent confirmation of human-to-human transmission of coronavirus on January 20. This suggest that at this point, markets believed that the risk of transmission to Europe was very low or that the European health system would be able to cope. However, there is a drastic change in the market’s response when Italy imposed its own quarantine on February 22. At this point COVID-19 had also spread to other Asian countries and many European countries had reported COVID-19 cases. We note from Fig. 1 that the decline was greatest for oil and gas perhaps highlighting the importance of the Russia-Saudi Arabia oil price war that started on March 8. For all the indices, the *CAR* plot starts to go upwards towards the end of March in response to the passage of the stimulus bill in the US Senate on March 25, 2020.

For the rest of the analysis, we sort the industries using the two-digit NACE industry classification. NACE is the European standard classification of productive economic activities.

## Results

We start by presenting results for between-industry differences in performance before analyzing within-industry differences. Table 2 shows the cumulative average abnormal returns across industries. Consistent with the expected impacts, all the industries were negatively affected by COVID-19 (column 1 of Table 2). From Table 2, we note that many of the largest significant declines are in industries that are relatively carbon intensive. Crude Petroleum Extraction, Basic Metals and Air Transport (and Travel Agencies) performed significantly worse while Electricity and Gas Utilities and Coke and Refined Petroleum performed relatively better. Utilities and refineries tend to be more diversified with utilities having a growing fraction of non-fossil sourced electricity while some refineries are also beginning to operate with biological material as their feedstock. In addition, utilities benefitted from continued demand for electricity during the crisis while air traffic volumes collapsed for example. Water Transport (sea and coastal passenger water transport) is also significantly affected. Pharmaceuticals, Food Manufacturing and Tobacco Manufacturing are some of the least affected industries, which again appears intuitively reasonable. From Table 2, we also note that the effect of the pandemic on stock returns is significantly reduced when we use the CAPM and the three-factor models.

**Table 2:** Stock market cumulative average abnormal returns (%) by industry

NACE	Industry description	Obs	RAW	*t* test	CAPM-adj	*t* test	FF-adj	*t* test	Carbon intensities
			(1)		(2)		(3)		(4)
6	Crude petroleum extraction	3	− 68.26	− 2.848***	− 23.00	− 1.623	− 12.34	− 0.929	–
79	Travel agency	1	− 67.96	− 3.145***	− 29.05	− 1.768*	− 16.67	− 1.059	0.05
50	Water transport	2	− 60.55	− 5.067***	− 17.18	− 1.852*	− 8.52	− 1.006	4.06
9	Mining support services	5	− 57.96	− 4.138***	− 20.67	− 2.041**	− 8.98	− 1.032	–
51	Air transport	5	− 57.81	− 4.227***	− 17.07	− 1.514	− 8.55	− 0.789	3.69
60	Programming and broadcasting	2	− 57.25	− 4.241***	− 44.67	− 4.106***	− 31.31	− 3.233***	–
59	Motion picture, video and sound	3	− 53.46	− 6.256***	− 47.59	− 7.447***	− 44.55	− 7.091***	0.03^a^
69	Legal and accounting	4	− 51.24	− 5.789***	− 3.69	− 0.559	− 3.06	− 0.473	–
56	Food and beverage service activities	3	− 50.6	− 6.947***	2.37	0.390	1.30	0.221	–
73	Advertising	2	− 47.02	− 3.882***	− 5.66	− 0.620	− 4.67	− 0.514	0.03
24	Basic metals	6	− 46.88	− 3.375***	− 24.62	− 2.635***	− 14.37	− 1.809*	2.35
29	Motor vehicles	15	− 46.82	− 4.279***	− 26.12	− 3.963***	− 21.17	− 3.490***	0.04
80	Security and investigation	4	− 45.53	− 4.852***	− 10.57	− 1.558	− 11.82	− 1.764*	0.03^b^
77	Rental and leasing	2	− 45.26	− 4.109***	3.38	0.498	4.70	0.693	0.06
70	Activities of head office	1	− 44.64	− 3.826***	− 38.88	− 4.962***	− 34.96	− 4.567***	0.02^c^
45	Motor vehicles trade and repair	1	− 44.31	− 3.851***	− 50.00	− 5.336***	− 44.01	− 4.837	0.06
55	Accommodation	3	− 44.28	− 5.170***	3.64	0.588	6.92	1.145	0.05
64	Financial services	60	− 44.11	− 5.050***	− 13.59	− 2.793***	− 5.52	− 1.668*	0.01
52	Warehousing	8	− 43.47	− 6.477***	− 5.76	− 1.251	− 7.85	− 1.745*	0.13
78	Employment activities	2	− 41.31	− 3.830***	− 30.20	− 4.219***	− 24.93	− 3.623***	0.02
82	Office administrative activities	2	− 41.3	− 4.230***	− 12.60	− 1.885	− 14.76	− 2.230**	–
3	Fishing and aquaculture	2	− 41.05	− 3.262***	− 13.20	− 1.151	− 17.97	− 1.586	1.12
30	Other transport equipment	10	− 40.6	− 5.420***	− 3.75	− 0.905	− 5.91	− 1.455	0.03
41	Construction of buildings	9	− 39.6	− 4.045***	− 6.81	− 0.927	0.88	0.133	0.10
19	Coke and refined petroleum	10	− 38.62	− 5.132***	11.39	2.151**	14.63	3.050***	4.15
53	Postal and courier activities	3	− 38.23	− 3.586***	− 23.45	− 3.043***	− 18.59	− 2.496**	0.12
81	Services to buildings and landscape	2	− 36.07	− 3.405***	− 12.52	− 1.392	− 15.16	− 1.708*	–
22	Rubber and plastic	6	− 35.77	− 3.604***	− 16.24	− 2.547**	− 11.40	− 1.887*	0.12
65	Insurance	32	− 35.19	− 5.504***	1.85	0.532	5.38	1.683*	0.05
7	Mining of metal ores	8	− 34.54	− 3.263***	− 1.60	− 0.227	4.15	0.644	1.42
90	Creative, arts and entertainment	1	− 33.65	− 3.033***	6.53	0.644	0.97	0.100	0.04^d^
27	Electrical equipment	7	− 33.52	− 3.920***	− 21.66	− 4.075***	− 20.20	− 3.873***	0.11
23	Other non-metallic mineral products	8	− 33.38	− 4.380***	− 6.64	− 1.605	− 4.39	− 1.090	2.79
46	Wholesale trade	7	− 32.56	− 4.078***	− 3.45	− 0.698	− 2.04	− 0.421	0.07
71	Architectural and engineering activities	7	− 31.62	− 3.572***	− 23.42	− 4.409***	− 24.46	− 4.701***	0.03
31	Furniture manufacturing	1	− 31.27	− 2.200**	− 5.96	− 0.473	4.57	0.386	0.04
58	Publishing	16	− 30.61	− 3.858***	− 9.08	− 1.774*	− 10.55	− 2.307**	0.02
68	Real estate	32	− 30.27	− 5.622***	10.21	2.265**	10.01	2.295**	0.004
28	Machinery and equipment	32	− 30.11	− 3.463***	− 14.23	− 3.474***	− 12.32	− 3.048***	0.05
47	Retail trade	31	− 28.83	− 4.056***	− 10.84	− 2.742***	− 8.39	− 2.206**	0.07
14	Apparel manufacturing	4	− 28.79	− 2.942***	− 1.56	− 0.224	− 7.45	− 1.152	0.11^e^
42	Civil engineering	6	− 27.94	− 4.395***	13.55	3.634***	11.36	3.172***	–
15	Leather	3	− 27.36	− 2.670***	− 9.05	− 1.436	− 12.86	− 2.121**	–
11	Beverage manufacturing	11	− 27.33	− 4.303***	8.78	1.788*	1.38	0.358	–
66	Auxiliary financials	22	− 27.1	− 3.377***	− 2.25	− 0.504	1.09	0.256	0.02
92	Gambling and betting	3	− 26.6	− 2.053**	9.52	0.901	14.04	1.345	–
20	Chemicals manufacturing	25	− 26.08	− 3.435***	− 15.51	− 4.840***	− 14.52	− 4.595***	1.04
43	Specialised construction	1	− 25.7	− 2.190**	7.61	0.720	9.04	0.887	–
62	Computer programming	5	− 25.31	− 2.267**	− 15.07	− 1.923*	− 19.84	− 2.738***	0.01
61	Telecommunications	21	− 24.25	− 4.059***	− 0.67	− 0.163	− 0.68	− 0.165	0.01
26	Computer manufacturing	21	− 23.51	− 2.854***	− 3.62	− 0.823	− 4.48	− 1.076	0.03
86	Human health	6	− 23.29	− 2.084**	− 15.37	− 1.925*	− 16.12	− 2.073**	0.03
87	Residential care	1	− 22.77	− 1.983**	− 4.36	− 0.436	− 11.76	− 1.253	0.04
35	Electricity and gas	24	− 21.21	− 3.509***	20.56	4.362***	16.44	3.746***	4.69
49	Land and pipeline transport	2	− 20.72	− 2.833***	0.53	0.091	1.75	0.303	0.72
17	Paper	13	− 19.54	− 2.631***	0.84	0.179	0.30	0.065	0.70
63	Information service activities	11	− 18.72	− 2.429**	0.95	0.176	− 1.23	− 0.243	0.02
36	Water	5	− 18.23	− 2.019**	9.58	1.241	9.62	1.268	0.26
72	Scientific research and development	10	− 16.58	− 2.015**	9.11	1.470	3.25	0.588	0.02
10	Food manufacturing	10	− 15.72	− 2.900***	10.80	2.668***	7.12	1.980**	0.24^f^
32	Other manufacturing	15	− 15.2	− 2.186**	2.75	− 0.498	− 1.96	− 0.460	–
12	Tobacco manufacturing	3	− 12.67	− 1.200	9.13	0.991	7.42	0.808	–
21	Pharmaceuticals	15	− 8.26	− 1.296	1.27	0.283	− 5.20	− 1.481	0.06
	All industries	595	− 31.64	− 5.153***	− 5.04	− 2.943***	− 3.74	− 2.297**	

This table presents cumulative raw returns (RAW), CAPM-adjusted (CAPM-adj) and Fama–French-adjusted (FF-adj) cumulative abnormal returns for each industry using the two-digit NACE industry classification. The sample consists of STOXX 600 index constituents as of December 31, 2019. The estimation window includes the months January to December 2019 while the event window starts from January to March 2020. The *t*-statistics are computed using the crude dependence adjustment (CDA) procedure presented in Brown and Warner (1980, 1985). The CDA test statistic accounts for cross-sectional correlation due to event clustering

The last column shows 2017 GHG emission intensities for each of the industries. The emission intensities are for 27 European Union countries and the data is obtained from Eurostat

*p < 0.10; **p < 0.05; ***p < 0.01

^a^Includes emissions from programming and broadcasting activities

^b^Includes emissions from office administrative and support activities

^c^Includes emissions from legal and accounting activities

^d^Includes emissions from gambling and betting activities

^e^Includes emissions from manufacturing of leather and related products

^f^Includes emissions from manufacturing of beverages and tobacco products

One important issue which confronts policy makers in many countries is which industries to help and under what conditions. There have been numerous arguments against bailing certain sectors such as airlines without any green conditionalities (see for example, Khan 2020; Wockner 2020). The result that relatively dirty industries performed badly during COVID-19 is thus important from a policy perspective. While it may be sensible for policy makers to want to compensate or help all the industries, many decision makers have seen an opportunity to impose green conditions on relatively dirty industries. An example is the French and Austrian governments, which have demanded that Air France and Austrian Airlines fulfill a series of tough restrictions in return for state support in connection with the pandemic.^11^ Within the dirty industries, it is possible that some of the companies may be relatively cleaner and therefore it will be less costly for them to comply with green conditions. In that case, they may be motivated to become even more greener. Some observers however, question whether environmental conditions should only apply to companies that receive support and argue that such rules should be general.

### Cross-Sectional Determinants of Abnormal Returns

The results in Table 2 (column 1) show that all industries responded negatively to the pandemic but that carbon intensive industries are among the industries with the largest negative abnormal returns while sectors that are less carbon intense have been much less affected. Obvious examples include food and pharmaceuticals. In this section, we focus on within-industry differences and investigate whether investors penalized companies with high carbon intensities or weak carbon-specific commitments. Tables 3, 4 and 5 present the results from the cross-sectional regressions. The dependent variables are the cumulative raw returns and CAPM-adjusted returns over the event period. Results using the Fama–French-adjusted returns are shown in Table 7 in the “Appendix”. We report standard errors adjusted to account for clustering at the country level. Since we have few countries (see Table 6) and industries,^12^ we follow the cluster bootstrap approach proposed by Cameron et al. (2008) to make finite-sample adjustments for the number of clusters. We focus on the models that include both industry and country fixed effects since they have significantly more explanatory power. From Table 3 (column 3 and 4), we note that the carbon intensity of the firm has significantly negative effects on abnormal returns during the coronavirus pandemic. For example, the results in column (3) indicate that a one-standard-deviation increase in carbon intensity lowers a firm’s *CAR* by 2.2 percentage points.^13^ This result indicates that “dirtier” firms fared worse. As for other control variables, we find that bigger and more profitable firms performed much better possibly due to their ability to absorb the COVID-19 shock better. The firm’s debt level has a positive effect on abnormal returns during the pandemic.

**Table 3:** Carbon intensity and stock returns

	RAW	CAPM-adj	RAW	CAPM-adj
	(1)	(2)	(3)	(4)
Carbon intensity	− 2.56***	− 0.35	− 2.84***	− 1.21**
	(0.556)	(0.783)	(0.611)	(0.510)
Firm size	2.29**	4.46***	2.03**	5.05***
	(0.931)	(1.222)	(0.876)	(0.885)
Profitability	0.15**	0.049	0.098**	0.055
	(0.056)	(0.056)	(0.035)	(0.063)
Leverage	− 0.065	− 0.014	0.043	0.024
	(0.101)	(0.063)	(0.049)	(0.042)
Constant	− 84.9***	− 108.6***	− 79.0***	− 122.5***
	(21.826)	(28.965)	(20.370)	(21.024)
Observations	579	579	578	578
Adjusted *R*^2^	0.140	0.101	0.230	0.220
Industry fixed effects	No	No	Yes	Yes
Country fixed effects	Yes	Yes	Yes	Yes

This table shows results from OLS regressions of individual stock abnormal returns on *Carbon Intensity* and firm characteristics. The dependent variables are cumulative raw returns (RAW) and CAPM-adjusted (CAPM-adj) returns. The event period for estimating the abnormal returns is January to March 2020. The variable *Carbon Intensity* is represented by GHG emissions normalized by the firm’s market capitalization on the day before the event window. GHG emissions are measured as the sum of Scope 1 and 2 emissions. The variable *Firm Size* is the natural log of market capitalization for firm *i* in industry *j*, *Leverage* is financial leverage calculated as average total assets divided by average total common equity and *Profitability* is the return on assets—a measure of a firm’s profitability. The sample consists of the STOXX 600 index constituents. Standard errors in parentheses are clustered at the country level

*p < 0.10; **p < 0.05; ***p < 0.01

**Table 4:** Climate change policy and stock returns

	RAW	CAPM-adj	RAW	CAPM-adj
	(1)	(2)	(3)	(4)
Climate change policy	− 4.34*	− 1.14	− 3.90*	− 1.86
	(2.279)	(1.620)	(2.212)	(2.257)
Firm size	2.76**	4.61***	2.58**	5.32***
	(0.955)	(1.257)	(0.962)	(1.086)
Profitability	0.14**	0.042	0.090**	0.046
	(0.054)	(0.060)	(0.032)	(0.068)
Leverage	− 0.050	− 0.0062	0.062	0.045
	(0.096)	(0.062)	(0.043)	(0.031)
Constant	− 93.1***	− 111.7***	− 89.6***	− 128.0***
	(22.673)	(29.518)	(22.089)	(24.489)
Observations	579	579	578	578
Adjusted *R*^2^	0.134	0.095	0.224	0.211
Industry fixed effects	No	No	Yes	Yes
Country fixed effect	Yes	Yes	Yes	Yes

This table shows results from OLS regressions of individual stock abnormal returns on *Climate Change Policy* and firm characteristics. The dependent variables are cumulative raw returns (RAW) and CAPM-adjusted (CAPM-adj) returns. The event period for estimating the abnormal returns is January to March 2020. *Climate Change Policy* is a dummy variable equal to 1 for firms that have outlined their intention to help reduce global emissions of the GHGs through their ongoing operations or the use of their products and services, and zero otherwise. The variable *Firm Size* is the natural log of market capitalization for firm *i* in industry *j*, *Leverage* is financial leverage calculated as average total assets divided by average total common equity and *Profitability* is the return on assets—a measure of a firm’s profitability. The sample consists of the STOXX 600 index constituents. Standard errors in parentheses are clustered at the country level

*p < 0.10; **p < 0.05; ***p < 0.01

**Table 5:** Environmental performance and stock returns

	RAW	CAPM-adj	RAW	CAPM-adj
	(1)	(2)	(3)	(4)
Environmental performance	1.59	− 0.83	0.42	− 2.79
	(2.557)	(3.247)	(2.322)	(3.321)
Firm size	3.01**	5.53***	2.99**	6.31***
	(1.235)	(1.276)	(1.163)	(1.191)
Profitability	0.40**	0.11	0.31*	0.083
	(0.144)	(0.209)	(0.158)	(0.234)
Leverage	− 0.023	− 0.0092	0.070	0.059
	(0.090)	(0.069)	(0.049)	(0.054)
Constant	− 105.2***	− 135.5***	− 104.5***	− 153.5***
	(28.750)	(30.415)	(26.927)	(28.049)
Observations	455	455	453	453
Adjusted *R*^2^	0.173	0.116	0.274	0.230
Industry fixed effects	No	No	Yes	Yes
Country fixed effects	Yes	Yes	Yes	Yes

This table shows results from OLS regressions of individual stock abnormal returns on *Environmental Performance* and firm characteristics. The dependent variables are cumulative raw returns (RAW) and CAPM-adjusted (CAPM-adj) returns. The event period for estimating the abnormal returns is January to March 2020. *Environmental Performance* is a dummy variable equal to 1 for firms in the top quartile of environmental performance, and zero otherwise. The variable *Firm Size* is the natural log of market capitalization for firm *i* in industry *j*, *Leverage* is financial leverage calculated as average total assets divided by average total common equity and *Profitability* is the return on assets—a measure of a firm’s profitability. The sample consists of the STOXX 600 index constituents. Standard errors in parentheses are clustered at the country level

*p < 0.10; **p < 0.05; ***p < 0.01

A potential explanation for the fact that carbon-intense firms did not do well is perhaps because investors already predicted governments would impose green conditions for bailouts. Yet another explanation could be that dirtier firms are being punished by the market. Investors may have sold their assets at the onset of the crisis and are now rebuilding their portfolios through investing in green stocks. Investors are increasingly aware that assets within carbon intensive industries face the risk of turning into stranded assets should countries impose ambitious climate policies. Bolton and Kacperczyk (2020) find evidence suggesting that investors in dirtier firms are already demanding higher returns possibly to compensate for climate change risk.

One encouraging interpretation of the within-industry results is that policy makers should not shy away from promoting cleaner industries and renewables and introducing ambitious climate policies given that the evidence suggest investors are already pricing climate change risk. These results are in line with, for example, Ramelli et al. (2020) who present evidence showing that investors penalized “dirty” firms in response to the unexpected success of the Global Climate Strike in 2019. Increasingly, there is more evidence that the previously held view among business leaders that “ESG hasn’t gone mainstream” is becoming outdated (Eccles and Klimenko 2019). However, an important issue to point out is that in terms of economic significance, the effect of our variable *Carbon Intensity* on firm performance during the pandemic period does not appear to be very large in magnitude. This may suggest that while investors are beginning to consider metrics such as a firm’s carbon footprint, the risk that these assets will be stranded by climate policy in the near future still appear distant in the absence of more ambitious climate policy. Alternatively, the results could imply that decarbonization will be gradual and in such a case, policy consistency is even more important to consolidate gains.

In Tables 4 and 5, we use the Environmental, Social and Governance (ESG) variables to explain within-industry differences in firm performance. We note that the ESG variable *Environmental Performance* do increase the explanatory power of the regressions as measured by the adjusted *R*^2^ more than does *Carbon Intensity*. However, these results are not statistically significant except for *Climate Change Policy*, which is marginally significant in the specifications using cumulative raw returns in Table 4. This may suggest investors view the existing carbon-specific commitments as either insignificant or insufficient in reducing climate risk, or both. The increasing availability of more granular data on firm-level carbon emissions may however, mean that more and more investors are relying on this data. Indeed one could argue that we should not expect ESG variables to help explain firm performance if investors can observe the actual firm-level emissions. In that case, investors can always see how dirty a particular firm is without relying on the ESG performance metrics or statements made by the firms. However, the growth of the ESG data providers industry suggests that these metrics are widely valued.

Another possibility is that investors may feel that firms will not follow through their carbon-specific commitments, especially after the crisis as they restructure and seek to maintain competitiveness. In addition, rescue packages may target all firms regardless of carbon-specific commitments while some governments may even roll back environmental regulations or favor lax implementation of existing environmental regulations. An alternative explanation for the insignificant climate responsibility variables is that *Climate Change Policy* is a weak signal of a firm’s climate credentials. Seventy-five percent of the firms in our sample report having a policy to address climate change. It may therefore be that having a “climate change policy” is a need primarily felt by the “worst offenders”—i.e. most carbon emitting companies. If the existence of such a climate change policy is largely lip service, then this variable cannot be expected to have much predictive or explanatory power. This may suggest that firms may simply engage in greenwashing to bolster their climate credentials. For example, out of 42 companies within the crude petroleum extraction, air transport, electricity and gas utilities, and coke and refined petroleum industries, 40 of them report having a climate change policy.

The growing interest in sustainable, climate friendly or green investments suggests that we really need a way to help fund managers avoid the biggest risks—and find the most appropriate green investment opportunities. If ESG performance metrics fail to provide the required information to investors, then there is an opportunity here for researchers to provide better information regarding the climate sensitivity of different industries. We also think it would be worth exploring another kind of index: If we estimate the kind of models we have here for say the Paris climate agreement or the 2016 US election, then the stock price sensitivity to these important events might provide a tentatively valuable indicator. The suggested index involves using the historical investor reactions to inform the current investor reactions. The idea then is to use this indicator in place of the standard ESG metrics since it contains more meaningful information on how investors view a firm’s climate initiatives and hence exposure to climate risk. Of course, companies adjust their strategies over time and previously dirtier firms invest in cleaner technology. In addition, each event is unique thus making comparisons across events difficult. However, such an index would be better as it incorporates actual investor reactions to related events.

### Containment Policies and Stock Returns

The relationship between stock returns and carbon intensity over the pandemic period depends on the stringency of containment measures in each of the countries. Countries adopted different policies initially designed to stop the transmission of the disease with the focus eventually turning towards dampening its spread across the population. We therefore explore the effect of different levels of containment policies on stock returns. We do so through interacting a measure of national lockdown stringency with firm-level carbon intensity. Stringency of containment policies is measured by the Stringency Index developed by Hale et al. (2020) which records the strictness of ‘lockdown style’ policies that primarily restrict people’s behaviour. From columns (2) and (3) of Table 8 we note that the interaction term is positive and statistically significant implying that the higher a firm’s carbon intensity, the greater (more positive) the effect of containment policies on stock returns. We note however, that these interpretations should be treated with caution. Many firms have production in multiple countries whilst the structure of our analysis assumes they are only active in one. This problem is due somewhat to the fact that many companies locate their headquarters in the UK for a series of reasons. The UK has, according to Table 6, 150 companies—a quarter of the total or the same number roughly as France and Germany combined. This does not accurately reflect the activity of these industrial countries since France and Germany together have much more manufacturing than the UK. In addition, there is an effect of firm composition. The UK actually has more firms in the financial services sector (banks and insurance companies). We find that including industry fixed effects in the regressions in Table 8 makes both the main effects as well as the interaction effect largely insignificant suggesting that some of the results are due to heterogeneous responses from different industries.

### Robustness Checks

To ensure the robustness of our findings, we carry out a number of sensitivity analyses in this subsection. Table 9 in the “Appendix” presents results with the standard errors adjusted to account for clustering at both the industry and country level. The results are largely unchanged and it appears we gain some precision through using two-way cluster-robust standard errors in some of the specifications. Our sample so far has included the financial industry. However, carbon emissions do not depict the full exposure of these firms since most of their exposure is through their lending activities. Recently banks have sought to limit this exposure by restricting lending to coal mining and new coal power plants. We therefore re-estimate our models while excluding the financial industry and the results remain unchanged. Finally, for now we have presented the cross-sectional analysis using the sum of Scope 1 and 2 emissions. We estimate the models again using Scope 1 and Scope 2 emissions separately. Our main variable of interest largely becomes insignificant when using a *Carbon Intensity* measure that consist of Scope 2 emissions. The results using Scope 1 emissions are largely similar to our baseline results in Table 3 suggesting that the results is largely driven by Scope 1 emissions.

## Conclusion

Policy makers will be disbursing very large funds, at least in the wealthier countries, to restart their economies. There are several strong arguments for a green profile on these investments. The urgency of the climate crisis is one and the risk of wasting scarce funds on projects that will soon turn out to be stranded assets is another—albeit related—reason. Our event study results shows that carbon intensive industries have been particularly affected during the COVID-19 crisis. However, that dirtier industries performed relatively worse does not automatically mean that they will not receive a lot of support. To the contrary, cheap oil and other fossil fuels may temporarily increase the attraction of fossil-based technologies. Fossil fuel firms are often large and concentrated and with the losses they have suffered, their lobbying activities are likely to be strongly reinforced. They have strong influence over many powerful political forces. Within industries, we find that prior climate-specific commitments and environmental performance as measured by ESG metrics does not help in explaining firm performance during this period. However, carbon intensity is associated with significant losses in the market value of firms during the first 3 months of the COVID-19 pandemic.

Many green investments have higher short run multipliers (require a lot of investment immediately) but low operating costs, low climate footprint and thus higher sustainability in the long run. Such investments should be the focus for the Green New Deals being discussed in various countries. Some of these are obvious like solar power or retrofits for buildings. However to identify, in general, which companies make good sustainable investments is difficult. Carbon intensity gives some useful information on firm performance during the pandemic while ESG related information appears less useful. We point to the possibility of using financial data to develop new indicators that could be used to guide investors.

## Appendix

See Tables 6, 7, 8 and 9.

**Table 6:** Stringency index and distribution of companies by country

Country	# of firms	Stringency index
Austria	9	19.41
Belgium	17	20.61
Britain	150	14.35
Cyprus	1	15.51
Denmark	21	18.29
Finland	17	18.44
France	84	26.42
Germany	72	19.93
Ireland	12	15.30
Italy	29	39.52
Luxembourg	6	17.01
Netherlands	29	15.08
Norway	14	20.22
Poland	8	16.63
Portugal	3	20.43
Spain	25	21.29
Sweden	45	6.17
Switzerland	53	18.56
Total	595

**Table 7:** Cross-sectional determinants of event period stock returns

Dependent variable	Fama–French-adjusted returns								
	(1)	(2)	(3)	(4)	(5)	(6)	(7)	(8)	(9)
Carbon intensity	0.47	− 0.10	− 0.10						
	(0.567)	(0.620)	(0.559)						
Climate change policy				1.42	0.69	0.69			
				(1.524)	(1.870)	(1.638)			
Environmental performance							− 0.84	− 1.94	− 1.94
							(2.733)	(3.087)	(3.169)
Firm size	3.07***	3.63***	3.63***	2.89***	3.53***	3.53***	3.77***	4.46***	4.46***
	(0.853)	(0.643)	(0.938)	(0.808)	(0.787)	(0.956)	(0.869)	(0.907)	(1.079)
Profitability	− 0.0022	0.035	0.035	− 0.0083	0.026	0.026	− 0.0087	0.028	0.028
	(0.066)	(0.066)	(0.073)	(0.069)	(0.069)	(0.079)	(0.173)	(0.191)	(0.216)
Leverage	0.058**	0.024	0.024	0.060*	0.037***	0.037**	0.045	0.039	0.039
	(0.026)	(0.015)	(0.024)	(0.031)	(0.011)	(0.016)	(0.030)	(0.028)	(0.032)
Constant	− 75.4***	− 88.4***	− 88.4***	− 72.3***	− 86.8***	− 86.8***	− 92.2***	− 108.3***	− 108.3***
	(20.172)	(15.296)	(21.844)	(19.203)	(17.690)	(21.662)	(20.594)	(21.348)	(24.962)
Observations	579	578	578	579	578	578	455	453	453
Adjusted *R*^2^	0.065	0.191	0.191	0.061	0.179	0.179	0.073	0.180	0.180
Industry fixed effects	No	Yes	Yes	No	Yes	Yes	No	Yes	Yes
Country fixed effects	Yes	Yes	Yes	Yes	Yes	Yes	Yes	Yes	Yes

This table shows results from OLS regressions of individual stock abnormal returns on *Carbon Intensity*, *Climate Change Policy*, *Environmental Performance* and firm characteristics. The dependent variables are the Fama–French-adjusted returns. The event period for estimating the abnormal returns is January to March 2020. The variable *Carbon Intensity* is represented by GHG emissions normalized by the firm’s market capitalization on the day before the event window. GHG emissions are measured as the sum of Scope 1 and 2 emissions. *Climate Change Policy* is a dummy variable equal to 1 for firms that have outlined their intention to help reduce global emissions of the GHGs through its ongoing operations or the use of its products and services, and zero otherwise. *Environmental Performance* is a dummy variable equal to 1 for firms in the top quartile of environmental performance, and zero otherwise. The variable *Firm Size* is the natural log of market capitalization for firm *i* in industry *j*, *Leverage* is financial leverage calculated as average total assets divided by average total common equity and *Profitability* is the return on assets—a measure of a firm’s profitability. The sample consists of the STOXX 600 index constituents. Standard errors in parentheses in specifications (3), (6) and (9) are clustered at the industry and country level while the rest of the standard errors are clustered by country

**p* < 0.10; ***p* < 0.05; ****p* < 0.01

**Table 8:** Carbon intensity, containment policies and stock returns

	RAW	CAPM-adj	FF-adj
	(1)	(2)	(3)
Carbon intensity	− 6.24**	− 11.7***	− 6.16*
	(2.593)	(3.365)	(3.214)
Stringency index	0.024	− 0.050	− 0.14
	(0.163)	(0.198)	(0.129)
Carbon intensity × stringency index	0.17	0.56***	0.32*
	(0.123)	(0.151)	(0.154)
Firm size	2.80**	4.65***	3.06***
	(1.039)	(1.318)	(0.922)
Profitability	0.15*	0.054	0.0051
	(0.076)	(0.049)	(0.054)
Leverage	− 0.12	− 0.059	0.036
	(0.114)	(0.069)	(0.027)
Constant	− 96.9***	− 111.9***	− 72.3***
	(27.952)	(35.617)	(24.041)
Observations	579	579	579
Adjusted *R*^2^	0.043	0.043	0.020
Industry fixed effects	No	No	No
Country fixed effects	No	No	No

This table shows results from OLS regressions of individual stock abnormal returns on *Carbon Intensity*, *Stringency Index* and firm characteristics. The dependent variables are cumulative raw returns (RAW), CAPM-adjusted (CAPM-adj) returns and Fama–French-adjusted returns. The event period for estimating the abnormal returns is January to March 2020. The variable *Carbon Intensity* is represented by GHG emissions normalized by the firm’s market capitalization on the day before the event window. GHG emissions are measured as the sum of Scope 1 and 2 emissions. The Stringency Index captures the strictness of ‘lockdown style’ policies that primarily restrict people’s behavior. The variable *Firm Size* is the natural log of market capitalization for firm *i* in industry *j*, *Leverage* is financial leverage calculated as average total assets divided by average total common equity and *Profitability* is the return on assets—a measure of a firm’s profitability. The sample consists of the STOXX 600 index constituents. Standard errors in parentheses are clustered at the country level

**p* < 0.10; ***p* < 0.05; ****p* < 0.01

**Table 9:** Cross-sectional determinants of event period stock returns

	RAW	CAPM-adj	RAW	CAPM-adj	RAW	CAPM-adj
	(1)	(2)	(3)	(4)	(5)	(6)
Carbon intensity	− 2.84***	− 1.21***				
	(0.240)	(0.359)				
Climate change policy			− 3.90**	− 1.86		
			(1.360)	(1.981)		
Environmental performance					0.42	− 2.79
					(2.037)	(3.520)
Firm size	2.03**	5.05***	2.58**	5.32***	2.99**	6.31***
	(0.886)	(1.195)	(0.928)	(1.234)	(1.086)	(1.356)
Profitability	0.098	0.055	0.090*	0.046	0.31***	0.083
	(0.057)	(0.075)	(0.050)	(0.081)	(0.086)	(0.237)
Leverage	0.043	0.024	0.062*	0.045	0.070	0.059
	(0.034)	(0.048)	(0.035)	(0.039)	(0.043)	(0.056)
Constant	− 79.0***	− 122.5***	− 89.6***	− 128.0***	− 104.5***	− 153.5***
	(20.436)	(28.025)	(21.452)	(28.381)	(25.143)	(31.608)
Observations	578	578	578	578	453	453
Adjusted *R*^2^	0.230	0.220	0.224	0.211	0.274	0.230
Industry fixed effects	Yes	Yes	Yes	Yes	Yes	Yes
Country fixed effects	Yes	Yes	Yes	Yes	Yes	Yes

This table shows results from OLS regressions of individual stock abnormal returns on *Carbon Intensity*, *Climate Change Policy*, *Environmental Performance* and firm characteristics. The dependent variables are cumulative raw returns (RAW) and CAPM-adjusted (CAPM-adj) returns. The event period for estimating the abnormal returns is January to March 2020. The variable *Carbon Intensity* is represented by GHG emissions normalized by the firm’s market capitalization on the day before the event window. GHG emissions are measured as the sum of Scope 1 and 2 emissions. *Climate Change Policy* is a dummy variable equal to 1 for firms that have outlined their intention to help reduce global emissions of the GHGs through its ongoing operations or the use of its products and services, and zero otherwise. *Environmental Performance* is a dummy variable equal to 1 for firms in the top quartile of environmental performance, and zero otherwise. The variable *Firm Size* is the natural log of market capitalization for firm *i* in industry *j*, *Leverage* is financial leverage calculated as average total assets divided by average total common equity and *Profitability* is the return on assets—a measure of a firm’s profitability. The sample consists of the STOXX 600 index constituents. Standard errors in parentheses are clustered at the industry and country level

**p* < 0.10; ***p* < 0.05; ****p* < 0.01

## Footnotes

1 See “How coronavirus stalled climate change momentum”, Financial Times: https://on.ft.com/3bsn2cv.

2 The French government for example, has set out a number of green conditions for supporting Air France that require halving of its overall emissions from domestic flights. See for example; https://www.flightglobal.com/strategy/french-government-sets-green-conditions-for-air-france-bailout/138160.article.

3 See https://www.cnbc.com/2020/04/20/oil-markets-us-crude-futures-in-focus-as-coronavirus-dents-demand.html.

4 See for example, *Bloomberg*, for evidence suggesting that greener firms may have outperformed dirtier firms during the pandemic. Laikola, Leo, ‘A $26 Billion Investor Says ESG Bets Outperformed in Selloff’, *Bloomberg*, June 15, 2020, https://www.bloomberg.com/news/articles/2020-06-15/a-26-billion-investor-says-her-esg-bets-outperformed-in-selloff, archive accessed on June 16, 2020.

5 Helm (2020) however, points out that Saudi Arabia’s decision to increase production following the collapse of talks with Russia came at a time when the pandemic’s economic impact was not yet fully regarded as serious.

6 It is of course of interest to judge the relative importance of these different mechanisms. Though COVID-19 disrupted global supply chains, the effect on European firms was not necessarily as large as sometimes suggested in the popular press. Initially when firms do not receive supplies of components, they draw down their inventories. In the case of COVID-19, the virus may have hit Europe before firms depleted their inventories—and in retrospect, the Chinese firms were not closed for a very long period. Companies like Volvo only stopped production in its Swedish and US plants from 26 March and this may have been partly to protect their workers and not only because of a lack of components.

7 See https://www.nytimes.com/interactive/2020/climate/trump-environment-rollbacks.html and https://environmentalintegrity.org/trump-watch-epa/regulatory-rollbacks/.

8 Jagannathan and Wang (1996) propose the conditional CAPM or C(CAPM) where the regression coefficients are conditioned on some global information set. While they show the C(CAPM) model yields greater accuracy compared to the conventional unconditional CAPM, one challenge lies in identifying the relevant conditioning relationships.

9 We control for country fixed effects given that the intensity of COVID-19 is heterogeneous across countries and almost all the response has been at the country level. In addition, climate policies at the country level also differs with countries such as Sweden having more ambitious climate policies in the form of a substantial carbon tax.

10 See http://mba.tuck.dartmouth.edu/pages/faculty/ken.french/data_library.html.

11 See https://www.euractiv.com/section/aviation/news/the-green-compromise-in-the-austrian-airines-bailout/.

12 Since the number of firms per industry is relatively small (see Table 2), we use the first level of NACE industry classification identified by an alphabetical code for industry fixed effects. This groups firms in our sample into 16 sectors.

13 This number is obtained by multiplying the standard deviation of the carbon intensity measure by the estimated coefficient.
